# Acquired Dyslexia in Spanish: A Review and Some Observations on a New Case of Deep Dyslexia

**DOI:** 10.1155/2005/872181

**Published:** 2006-01-09

**Authors:** Robert Davies, Fernando Cuetos

**Affiliations:** The University of OviedoSpain

**Keywords:** Dyslexia, deep dyslexia, phonological dyslexia, lexical, grapheme, phoneme, Spanish

## Abstract

Readers and writers of Spanish use an orthography that is highly transparent. It has been proposed that readers of Spanish can rely on grapheme-phoneme correspondences, alone, to access meaning or phonology from print. In recent years, a number of case studies have yielded evidence inconsistent with this idea. We review these studies with particular focus on those that report evidence for reading based on direct lexical mappings between print, orthographic representations, and meaning or phonology. We report a new case of acquired literacy impairment in Spanish, MJ, who presents a pattern of preserved abilities and deficits symptomatic of deep dyslexia. The patient is unable to read nonwords, but can read a substantial number of words. Her reading is characterized by the production of semantic, visual, and derivational errors. We argue that MJ has a deficit in her lexical selection ability, common to both her reading and her naming problems. We propose that MJ, and the other cases we review, demonstrate that lexical reading is adopted by skilled readers even in a transparent language.

## 1. Introduction

Readers of Spanish enjoy a largely transparent alphabetic system. Orthography is reflected in phonology in a rule governed fashion. It has been claimed that, for this reason, knowledge of grapheme-phoneme correspondences (GPCs) alone is sufficient to access meaning or phonology in Spanish [1–3]. In the present article, we review previous reports of acquired reading impairment in Spanish. We then report a new case of acquired dyslexia, a Spanish-speaking patient who presents a pattern of impairments and preserved abilities that can be characterized as deep dyslexic. We argue that these cases open a window on the factors that motivate the use of direct lexical mappings in reading, and thus contribute essentially to our understanding of literate behaviour in general. To anticipate, it is our view that the relative transparency of an orthography may not seem to warrant the use of lexical mappings as well as spelling-sound correspondences, but that such knowledge can nevertheless be critical in reading or writing where it is afforded by experience, or demanded by the circumstances of a task.

Ardila [1,2] argues that for readers of an alphabetic language like Spanish, which embodies grapheme-to-phoneme correspondences with few or no inconsistencies [1, p. 444], "Reading ... is always mediated by phonology ... " That is to say, if a language community employs an alphabet with largely or entirely predictable GPCs, then the speakers of that community will not use direct mappings from graphemes to the lexicon to perform literate behaviours. In reading a transparent language, the correct pronunciation will always be found for known and unknown words using sublexical mappings between graphemes and phonemes. The meaning of words will always be accessed through mappings from graphemes to phonemes, and then from phonemes to the entries of the lexicon. Where reading problems arise, one would not therefore expect to find

ISSN 0953-4180/05/$17.00 © 2005 – IOS Press and the authors. All rights reserved errors in which the target printed word is read as a semantic relative, e.g. "dog" → <<horse>>, a semantic paralexia. (Note that in the present article we report printed stimuli in lower case letters in quotation marks, and spoken responses or auditory stimuli in lower case letters between angled brackets.) Consistent with this claim, Ardila and colleagues [1,3] report the complete absence of semantic paralexias in the errors produced in tests of reading, in group studies of Spanish-speaking aphasic patients. Yet, these authors allow that such a null observation could be due to the small size of the sample employed in their studies, and it will be seen that this is a possibility that must be re-examined.

In the following, we review a small number of cases of acquired impairments in Spanish that present strong evidence for the use of direct lexical mappings in reading or spelling words of a transparent orthography. We focus on cases which have been described as presenting the symptoms of acquired deep dyslexia, and of phonological dyslexia. It is worth rehearsing the critical features used to make these attributions. We will not refer in detail to a further type of dyslexia, surface dyslexia, and the interested reader is directed to the article by Ferreres and colleagues, in this issue.

In a systematic review of 22 deep dyslexic patients, Coltheart [10] concludes that the observation of semantic paralexias in reading is central to the characterization of a patient as deep dyslexic. He goes on to argue that the occurrence of a number of further symptoms are consistently associated with the appearance of semantic errors. Among these, one can expect to record in a test of reading aloud the occurrence of visual errors e.g. "gallant" → <<gallon>>, function word substitutions e.g. "for" → <<and>>, and derivational errors e.g. "sickness" → <<sick>>. The patient will tend, also, to be more often correct in reading highly imageable compared to less imageable words, and will find verbs harder to read than adjectives and adjectives harder to read than nouns. In general, they will have more difficulty reading function words than content words. They can also be expected to present an impairment of writing, whether spontaneous or to dictation. And they will be likely to present evidence of an impairment of auditory-verbal short-term memory. One can expect to find, lastly, that the patient will find it impossible to read aloud a nonword or pseudoword.

The observation of an impairment of nonword reading is critical for the discussion of lexical reading. For as will be seen, in at least one major account of acquired dyslexia [11], nonword reading is taken to be possible only by using sub-lexical grapheme-phoneme correspondences. Therefore, if a patient demonstrates an ability to read words, but a relatively impaired ability to read nonwords, then they may be argued to employ lexical rather than sublexical knowledge in their reading of words.

Phonological dyslexia is a term used to characterize patients who present a subset of the symptoms of deep dyslexia [16]. The critical observation is that the patient presents not a complete loss of their ability to read nonwords but an impairment relative to their ability to read words. In addition, they are not expected to produce semantic errors, though they can be expected to produce visual errors of the kind exemplified above. In some cases, but not others, they may be observed to demonstrate, also, an advantage in reading more imageable compared to less imageable words, they may present an effect of grammatical class, and they may also produce derivational errors in their reading performance [20,41].

It has been argued by Friedman [21,23] that, in fact, deep dyslexics are severe cases of phonological dyslexia. Friedman [21,23] reports analyses of patients who were initially recorded as presenting the criterial symptoms of deep dyslexia, especially the production of semantic paralexias and an almost total inability to read nonwords. These patients, after a period of some months following the initial assessments, were found to show improved word and nonword reading, and were observed to produce few, if any, semantic paralexias. The key point is that the symptoms observed in cases of deep and of phonological dyslexias, whether or not they are in fact separable syndromes, suggest the idea that direct lexical mappings can be used in reading.

Coltheart and colleagues [11] propose a Dual Route Cascaded model of reading which accounts for the symptoms presented in cases of phonological or deep dyslexia, together with the symptoms presented by a third group of patients, characterized as surface dyslexics. Reading aloud can be done using grapheme-phoneme correspondences, some of which might be modified by context-specific variations. The outputs of these mappings are blended or assembled to create phonological forms in response to print. Such mappings are sufficient to read aloud both nonwords and words in a transparent language like Spanish. They are sufficient to deliver some but not all correct pronunciations in a language like English, however. For many words in English are irregularly pronounced e.g. "pint" is pronounced /pInt/ but "mint" is pronounced /mint/ (transcription of phonemes in the International Phonetic Alphabet are shown between forward slash marks). Thus readers of English are claimed to require, also, direct mappings from graphemes to unitary representations of lexical orthography and then to representations of lexical phonology to serve the pronunciation of irregular exception words. Exception words can also be pronounced through mappings that traverse a pathway from lexical orthography to lexical semantics.

In this scheme, phonological dyslexia is attributed to an impairment of a patient's ability to complete grapheme-phoneme mappings. As a result, the patient can read words, including irregular exception words, but not nonwords. Deep dyslexia is explained by an impairment not only of the grapheme-phoneme mappings but also of the patient's ability to complete mappings by the direct lexical route. The deep dyslexic cannot read nonwords and produces errors in reading words that suggest a deficit in the adequacy of direct lexical mappings e.g. semantic paralexias. Surface dyslexia arises when the direct mappings are impaired but the grapheme-phoneme mappings are preserved so that in a language like English the patient can read regularly pronounced words and nonwords but is impaired in her ability to read exception words. In principle, this model can be used to account, also, for symptoms of acquired disorders of writing, e.g. surface, phonological or deep dysgraphias [17].

The dual-route model of reading was designed to account for normal and impaired reading in a quasi-regular language like English (see, also, articles by Plaut, McClelland, Seidenberg and Patterson [36,42] for a different view). In the present article, we discuss whether it is equally applicable to explain patterns of reading in a largely regular language like Spanish. What are the characteristics of spelling-sound correspondences in Spanish? Literate speakers of the language employ an orthography with highly predictable mappings from graphemes to phonemes, and from phonemes to graphemes. The majority of graphemes have a context independent pronunciation. (We discuss normative pronunciations or spellings in Spanish, as used in Spain, only.) In the case of "c", "r", "g, "y", and "h", some variation obtains across different lexical contexts but correct pronunciations can be predicted in a rule-governed manner, in relation to the neighbouring graphemes in a word. For example, the letter "r" is usually read as /r/ but it must be read as /rr/ if it appears at the start of a word, or after the letters "n" e.g. "enredo", "s" e.g. "Israel", and "l" e.g. "alrededor".

In addition, stress is lexicalized and must be recognized where it is marked graphically by an accent. In the regular, or type majority, pattern the stress in a word is assigned to the penultimate syllable e.g. "ventana" is read as <<venTAna>> (the stressed syllable is capitalized). For two groups of words, however, the stress is either assigned to the last syllable e.g. "corazón" is read as <<coraZON>>, not <<coRAzon>>, or it is assigned to the syllable before the penultimate syllable e.g. "lámpara" is read as <<LAMpara>> not <<lamPAra>> or <<lampaRA>>. Moreover, for some pairs of words, differences in meaning are indicated by the presence of a stress marker, e.g. "sabana" signifies a savannah but "sábana" refers to a sheet.

The majority of phonemes in Spanish are spelled according to single phoneme-grapheme correspondences. There are a small number of phonemes, however, that have different possible spellings in Spanish. Variations in spelling are either arbitrary, or semi-regular such that a phoneme's spelling can be predicted by phonemic context in some but not all of the phoneme's appearances. The phoneme /x/ before /e/ and /i/ can be written as "g" e.g. "gente" or "j" e.g. "jefe". The phoneme /k/ is spelled as "qu" before the letters "e" e.g. "queso" or "i" e.g. "quien", with "c" before the letters "a" e.g. "casa", "o" e.g. "costa", or "u" e.g. "cuarto" but it can also be spelled with a "k" irrespective of proximal graphemes, e.g. for words like "kilo". The phoneme /b/ can be written either as "b" or as "v". The letter "h" is always silent, thus what might be termed a zero phoneme is either spelled with an "h" e.g. "zanahoria", or is left without an orthographic presence. The phoneme /j/ can be spelled as "ll" e.g. "lluvia" or as "y" e.g. "yegua".

Thus, it is possible to conclude that one could correctly read aloud the majority of words in Spanish using only grapheme-phoneme correspondences, in some cases modulated by context-specific rules. The majority of spellings in Spanish also appear to be achievable using sub-lexical correspondences. However, the arbitrary nature of certain spelling variations, together with the fact that the words that contain inconsistently spelled phonemes are often very frequent, mandate that writers of Spanish must learn orthographic conventions [25]. The analysis of spelling errors in Spanish children by Valle-Arroyo [46], as well as the report by Cuetos [12] of lexical priming of adult spelling, certainly indicates the use of orthographic lexical knowledge. At this point, then, there appears to be no reason to expect the evidence of lexical reading, but some basis for the expectation of lexical spelling in Spanish.

It is striking, therefore, that a number of cases have been reported in which, following brain injury, speakers of Spanish have produced reading or spelling behaviours that can be explained by the use of lexical reading or spelling strategies. Of particular importance has been the observation of semantic errors in the reading of words, in some cases, and the finding in other cases of substantial word reading abilities preserved alongside a relative impairment of nonword reading. We review these cases below, but we acknowledge at the outset that a critical review by Ardila [2] argues that they are not relevant to the question of whether Spanish readers might use direct lexical mappings, to access semantics, or phonology, in normal reading. He proposes that the cases can be seen as exceptions, irrelevant to our understanding of normative reading in Spanish. This is because, firstly, semantic paralexias in Spanish are rarely observed following brain injury and, secondly, such errors had been observed (at the time of his review) in a rather circumscribed group of individuals who are claimed to possess characteristics that make it difficult for us to generalize to the literate Spanish population. All were male, highly educated, and had been the recipients of many months of speech therapy; some were multilingual. Ardila [2] argues that the individuals presenting acquired dyslexia whom we discuss below may have produced semantic paralexias either as a result of having adopted a strategy of lexical reading as a result of their therapy, or because they had learned to use direct lexical mappings through premorbid experience of reading in non-Spanish languages. We suggest in the following that, on the contrary, the cases must be seen as integral to our understanding of reading in Spanish.

The earliest report of acquired reading disorders in which, critically, the patients produced semantic errors in reading is presented by Ruiz and colleagues [40]. We can address, in regard to these patients, the question: Can speech therapy evoke patient adoptation of direct lexical mappings in reading? The patients are reported to have presented with symptoms of total alexia immediately post-injury but had arrived at the impaired yet substantial ability to read correctly, out of a test battery of 281 words, 211 (75%) in the case of ON, 183 (65%) in the case of MG. In these cases, at assessment, intensive speech therapy had been received for periods of between 6 and 18 months post-injury. Both patients were very well educated and had led highly literate lives prior to their injuries. We assume that word reading tests sample some fraction of the total reading vocabularies accessible to the patients. While it is true that it is unclear how much practice is required to develop a sight vocabulary in a healthy or in an impaired adult, we argue that it is more likely that the patients' extensive pre-morbid literacy underlies their reading strategies post-injury, than that such strategies originated substantially in therapy.

Did these patients offer evidence of the use of direct lexical mappings in reading? Both were largely unable to read aloud phonologically legitimate nonwords. Both produced verbal paralexias in response to word targets, of which, 15% (MG) to 23% (ON) were semantically but not formally related to the targets. Numeric trends suggested an advantage in the reading of high-compared to low-frequency or imageability nouns in the patients' reading. An effect of grammatical class was also evident in their performance. In sum, the patients ON and MG present, firstly, evidence of severely impaired grapheme-phoneme mappings and, secondly, evidence of a reliance on direct lexical mappings.

Ferreres and Miravalles [18] report the case of JMK who also produced semantic paralexias in tasks of reading aloud and, also, proved unable to perform a test of nonword reading. JMK had received 12 years of schooling. He had been multilingual, however: notably, speaking and writing Slovenian. Before testing, JMK had received up to 5 years of rehabilitation treatment post-injury. At assessment, JMK was found to be impaired in picture naming and in reading but not in auditory comprehension. Ferreres and Miravalles observed that whereas JMK was wholly unable to read nonwords he was able to read correctly 21 out of 250 (8.4%) of words presented in isolation. Most critically for our concerns JMKs errors in oral word reading were found to consist largely (48.4%) of verbal paralexias, of which, 57.3% were semantically related but formally unrelated paralexias.

We would concur with Ferreres and Miravalles that JMK's semantic errors were unlikely to have arisen because of reading strategies adopted as a result of his knowledge of other languages since the only other language he could write, Slovenian, possesses consistent grapheme-phoneme correspondences. The lengthy period in which JMK received therapy prior to testing allows the possibility, however, that the limited success he achieved in reading words depended upon orthographic patterns learned in that time. The high proportion of omissions (43% of responses) and the low number of correct responses presents a different picture to that seen in the cases of ON and MG. It seems to us arguable that JMK's reading could have depended upon a limited set of direct mappings acquired through therapy.

A more recent case is reported by Cuetos and Labos [14]. JD is a Spanish-speaking reader who presented a severe impairment of nonword reading, together with evidence of lexical reading. JD had received speech therapy over a period of two years at the time of his study. He showed some problems in oral and written comprehension but his performance could be interpreted as normal, or as falling at the low end of the normal range. Despite this, and though JD could repeat words well, he was severely anomic, presenting restricted spontaneous speech, and scoring very poorly in tests of reading aloud. His oral reading, also, was impaired and he produced few correct responses. Even at the low level attained in his reading, it was apparent that he found it very much harder to read verbs and function words compared to nouns. JD produced a number of semantic errors. As noted, he was wholly unable to read nonwords aloud.

Cuetos and Labos argue that JD's impairments were caused by problems located at the semantic level and at the phonological output lexicon. He was found to be worse in his spoken output compared to his written output of the same items. Moreover, he could understand many of the words he could not name. Thus, we suggest that JD presents further evidence that Spanish readers can adopt lexical strategies in reading. It is critical to our interests that, again, the patient had been a very skilled reader before his injury. Equally, it should be noted that he had received speech therapy for some months prior to assessment though, again, we acknowledge the uncertainty concerning how much therapy, and what kind, must be received to promote lexical reading in brain injured adults.

The four cases we have reviewed so far constitute all but two of the published reports of acquired deep dyslexia in Spanish, to the best of our knowledge. As has been noted, Ardila [1,3] did not find evidence for semantic paralexias in the reading of Spanish-speaking aphasics. Furthermore, in a review, Ardila [2] suggested that the cases of, MG and ON may not be relevant to understanding reading in Spanish because semantic paralexias are rarely observed. We would argue that semantic paralexias are rarely seen in impaired Spanish reading, indeed, that patients presenting symptoms consistent with deep dyslexia are rarely reported, not because such errors or such patients are abnormal but as a result of the requirements of reading in Spanish, and because of the narrow set of criteria by which the diagnosis can be affirmed. This can be seen in the cases we review next.

Cuetos [13] reports a patient who initially presented evidence of a reading impairment of the deep dyslexic type. PR had been well educated prior to her injury. At her first assessment, eight months after she had suffered a brain injury, PR was wholly unable to read nonwords but could read 158 (74%) words correctly out of a set of 214. She was more likely to read a word correctly if it was more imageable, or more frequent. She also found it easier to read nouns, adjective and (curiously) function words than verbs. Her errors were largely visual (24% of all errors), semantic (13%) or derivational (16%). At a second assessment, 26 months post-injury, PR was found to have recovered, substantially, her ability to read nonwords, and was able to read correctly 67% of the nonwords presented. In addition, she no longer made semantic errors. PR is thus consistent with the idea that patients who present deep dyslexic symptoms may suffer, in fact, from a severe form of phonological dyslexia, and that they may recover over time to present the less extensive symptoms of the latter condition. It is critical, moreover, that PR's recovered ability to read nonwords appears together with an absence of semantic paralexias in her reading. Later in this article, we discuss the possibility that phonological information delivered by grapheme-phoneme correspondences may serve to prevent the output of semantic errors in reading.

Evidence consistent with this idea is reported by Cuetos, Valle-Arroyo and Suarez [15] in a case study concerning a patient, AD, who presents evidence of a phonological dyslexia in Spanish. (Ferreres and colleagues [19] have reported another case of phonological dyslexia in Spanish, AP, but do not discuss his performance on tasks of lexical reading in detail.) AD, like the other patients we have reviewed, was a highly educated individual and had been extensively literate prior to his injury. The gap between the onset of his condition and the start of testing was no more than three months, however. During testing, it was found that he was impaired in reading words and nonwords, reading correctly 344 out of 386 (89%) words and 49 out of 141 (35%) nonwords. It is clear, however, that his nonword reading deficit is not as severe as the seeming total abolishment observed in the deep dyslexia cases. AD presented no effect of grammatical class, imageability or frequency in his oral reading of words.

Further investigation indicated that AD was able to repeat words and nonwords well, and that his visual lexical decision and his written comprehension was intact. This substantial preservation of the skills necessary to achieve direct lexical mappings, contrasts with the impairment of skills likely to be needed in nonword reading. AD was able to perform orthographic and phonological segmentation, he was near perfect in giving the sounds of letters but, crucially, he was severely impaired in his ability to blend phonemes. It should be noted that AD did not present a deficit in his verbal short-term memory. Though we regard short-term memory as a likely location for nonword assembly, the observation that he did not present an impairment of short-term memory implies that his nonword reading impairment was caused by a problem in a different resource also, presumably, required for blending phonological output. We suggest that the absence of semantic paralexias in AD's reading, together with the absence of imageability and other lexical effects, is due to the influence of intact GPC outputs. This possibility is discussed further, in the following.

We now turn to focus on the question of why semantic paralexias occur. Shallice and Warrington [43] suggest that such errors are produced if individuals suffer an impairment of their phonological reading ability, seen in a nonword deficit, alongside at least one other impairment. The latter might, in different cases, be located at the visual recognition process, at the access of semantics, within the semantic system, or at the process of phonological retrieval following semantic specification. Newton and Barry [35] report findings which suggest that less imageable words are harder to lexicalize under normal circumstances so that, in the absence of support from the outputs of grapheme-phoneme correspondences, such words are more vulnerable to error. This is seen in the imageability effect in deep dyslexic reading. The idea that less imageable words are harder to lexicalize receives support, also, in findings reported in relation to healthy adult reading [44,45]. It has been proposed that an intrinsic imprecision of semantic specifications may be exposed by the impairment of grapheme-phoneme mappings because the latter serve to correct or check the former before output [34]. In a transparent language like Spanish, relatively severe impairment of the phonological route may be required before semantic errors may be observed [48]. One can suppose that even if direct lexical mappings are used in reading in Spanish, reliance on such mappings will be less in comparison to that required for reading in a language like English, so that the existence of semantic paralexias will tend to be more rare in cases of acquired reading impairment in Spanish than is observed in English.

Yet Morton and Patterson [31] point out that some deep dyslexics produce semantic errors but demonstrate an awareness of their errors, as well as an ability to discriminate semantic paralexias from target responses. Thus, semantic paralexias may arise not because an intrinsically unstable, or imprecise, lexical-semantic route is exposed by the impairment of grapheme-phoneme reading, but because correct responses are blocked, leading to the selection of semantically related errors. If semantic paralexias occur as a result of lexical selection problems, rather than semantic problems, one might expect to find an associated problem of speech production e.g. an impairment of picture naming. Just such a case is VJ is reported by Laine and colleagues [29]. VJ presents impaired nonword reading, and a severe anomia in association with the production of semantic paralexias in reading.

It is unclear how lexical selection problems might occur. Recent investigations of speech production in healthy adults [8,9,38] support the conjecture, however, that short-term memory has a critical role in lexical selection so that its impairment could be associated with a lexicalization deficit. Of course, a short-term memory deficit is observed in cases of deep dyslexia, together with the production of semantic paralexias. We consider in more detail, in the Discussion, the link between deficits in short-term memory and the production of semantic paralexias in deep dyslexic reading, with particular reference to the case we now report.

## 2. The case study

MJ is a right-handed woman, and was 38 years old at the time of testing. Prior to injury she had worked as a secretary. In November 2003 she was admitted to hospital with a suspected tumour. Upon admission, a CAT scan indicated an area of reduced brain density in the left basal ganglia. An MRI scan confirmed the existence of a tumour of the astrocitomatic type in the area of the left hemisphere. In December 2003, an operation to perform a stereotaxic biopsy resulted in a brain haemorrhage. A second CAT scan, performed subsequent to the biopsy, indicated one lesion located in the thalamus, corresponding to the initial tumour, and one lesion in the striate cortex, corresponding to the effect of the haemorrhage. The attending neurologists initially characterized MJ as presenting evidence of a "mixed aphasia", with severe production problems, and some problems of comprehension. She continues to be paralyzed in her right arm and right leg.

In January 2004, MJ began a course of oral speech therapy which continued throughout the time of testing. In June 2005, she began to receive therapy aimed at improving her writing skills. MJ is now learning to write using her left hand. We note that the data we report were largely gathered in a period beginning in April 2005 and ending in June 2005. Thus, our evidence is unaffected by the writing therapy MJ has received. MJ presents a severe impairment of conversational ability for she is able to produce, repetitiously, only very simple phrases or sentences. This impairment no doubt stems from difficulties with grammatical construction, also observed, as well as her substantial anomia. MJ presents severe problems of reading and writing that can be characterized as dyslexia and dysgraphia, as will be seen. Yet she appears to retain a highly functioning capacity for auditory and visual language comprehension.

In the following, we report a series of tests designed to probe MJ's language abilities. We focus on the single word level, specifically, on recognition, oral reading, and the comprehension of printed words. We report, firstly, the findings of our study of visual word recognition ability, then on the results of tests of her comprehension of concepts, before going on to report our observations concerning her capacity for speech production and her reading skills. (In our discussions, where we consider how MJ compares with healthy adults, we refer to normative data where it is available.) To anticipate, MJ is severely impaired in her ability to transcode graphemes as phonemes and has substantial word finding difficulties but presents, alongside these problems, a preservation of phonological knowledge, comprehension ability and semantic knowledge.

### 2.1. *Visual word recognition*

We tested MJ's word recognition using a visual lexical decision task taken from the Spanish version of the PALPA [28], the EPLA produced by Valle and Cuetos [47]. A set of 40 items were presented and MJ was asked to indicate verbally whether the items were or were not real words: 20 were words; 20 were pronounceable nonwords created by substituting one or two letters in words. MJ performed well in the task, achieving a score of 36 (90%) responses correct. MJ's errors were made to one word, "ofensa" (offence) and three nonwords, "cafon, hucho, balenza". Extrapolating from the normative data provided for the test (since available data relate to a larger set of lexical decision items than those administered), healthy adults would be expected to correctly distinguish, at minimum, 19 out of 20 words and 18 out of 20 nonwords. We suggest that MJ presents a preserved visual word recognition ability.

### 2.2. *Semantic knowledge and verbal comprehension*

We tested MJ's verbal comprehension using two word-picture matching tasks. In a task drawn from the EPLA, MJ was asked to listen to a word and then to point to the single picture in a set of five pictures that matched the meaning of the word. The five candidate matches depicted concepts that were visually related, closely or distantly semantically related, or were unrelated to the named concept. MJ's responses were correct in 38 (95%) of 40 trials. One of her errors was to indicate a close semantic relative, (names for picture stimuli are shown in capitals) CAMISETA (undershirt) in response to the target <<calzoncillos>> (underpants), the other error was to point to a distant relative, ESPEJO (mirror) in response to the target <<peine>> (comb). A sample of healthy adults would be expected to achieve an average score of 39.45 items correct, with a standard deviation (s.d.) of 1.67. If we set the lower limit of normal performance at the average minus two standard deviations we can conclude that MJ's performance was normal in this first test. In a second task, devised for the study, we probed MJ's ability to discriminate close semantic relatives, asking her to indicate over a series of trials whether the concept labeled by a spoken word was or was not depicted in a probe picture. The spoken word was either the dominant name for the pictured object, e.g. the stimulus pair, TIGRE-<<tigre>>(tiger), or the name of a close semantic relative, e.g. the stimulus pair, CAMÍON(van)-<<coche>>(car). Again, MJ performed very well, producing 40 correct responses in 40 trials. The results of both tests demonstrate that MJ's ability to comprehend auditorily presented single words was preserved at a high level. To examine MJ's semantic knowledge more closely, we administered two additional tests.

Firstly, we used an instrument modeled on the Pyramids and Palm Trees Test [24]. MJ was given a target picture and was then asked to point out which one of two pictured concepts could be associated in meaning to the target. For example, MJ was required to indicate whether an APPLE or an ONION was semantically related or associated to a TREE. MJ responded correctly in 24 of 25 (96%) trials.

Secondly, we administered an auditory test of synonym judgment, taken from the EPLA. 60 pairs of words were read aloud to MJ. Half the pairs were very similar semantically, near-synonyms e.g. <<océano>> (ocean) and <<mar>> (sea), and half the pairs were semantically unrelated, control pairs e.g. <<convento>> (monastery or convent) and <<boda>> (wedding). The pairs were formed of words that were of either low or high imageability, with words matched on frequency across imageability conditions. MJ had to verbally indicate, <<si>> or <<no>>, whether the stimulus pairs consisted of words that were approximately the same or were different in meaning. She was correct in 53 out of 60 trials (88.33%). She produced only one incorrect response to word pairs consisting of high imageability words, to the control pair <<alegría>> (happiness) and <<cosecha>> (harvest, or yield). She produced six errors to low imageability pairs, however, one to the 'synonymous' pair, <<consejo>> (advice) <<sugerencia>> (suggestion) and five errors to the control pairs: <<mentira>> (lie) and <<suerte>> (luck); <<ventura>> (happiness) and<<debilidad>> (weakness); <<sugerencia>> (suggestion) and <<clemencia>> (mercy); <<reproche>> (reproach) and <<consejo>> (advice); and <<dolor>> (pain) and <<compasíon>> (compassion). Healthy adults could be expected to score, on average, 29.64 items correct for high imageability pairs (s.d. of 0.64), and 28 correct on low imageability pairs (s.d. of 2.41). MJ scored 29 correct on high imageability, and 24 correct on low imageability pairs. Therefore, if again we take the lower limit on normal performance in this task as the mean minus two standard deviations, MJ demonstrates a level of performance at least as good as that shown by healthy adults.

We conclude that MJ presents extensive preservation of her comprehension ability and of her semantic knowledge. Yet her speech is severely restricted. This suggests that her primary deficit may lie in the production of lexical responses. In the next section, we report findings yielded by tests of her ability to produce words in response to semantic information.

### 2.3. *Semantically-driven verbal production: Fluency and picture naming*

We examined MJ's speech production by testing verbal fluency and picture naming. The verbal fluency tasks can be said to draw on knowledge of semantic associations since the patient is asked to produce words in response to stimuli in the manner of the free association task, "What word(-s) comes to mind upon seeing some other word?" or the category-exemplar generation task, "What examples can you produce for a given category label?" [6,33]. Associative relatedness is highly correlated with semantic similarity for many pairs of words, but associative relatedness and semantic similarity have been found to be separable factors in tests of word recognition [32]. The picture naming task, however, most certainly requires a translation between representations at the semantic and lexical levels.

In the verbal fluency task, we asked MJ to respond to the spoken semantic category labels, <<frutas>> (fruits) and <<animales>> (animals), requiring her to produce as many words as she could think of, in reaction to these words, in one minute. MJ could produce only four names of animals and two names of fruits. We then asked her to produce as many words as she could, again in one minute, in response to the letters <<p>> and <<f>>. MJ was able to produce only one word to the letter <<p>> and she produced no responses to the letter <<f>>.

We tested MJ's object naming ability using the Spanish adaptation of the Revised Boston Naming Test [26], the "Test de Vocabulario de Boston" [27]. In this test, the target picture names are progressively harder to produce and the recommended procedure is to end the session when the participant has produced four incorrect responses in a row. We asked MJ to complete the full test and found that MJ correctly named 16 pictures (26.67%) out of a set of 60 targets.

In those instances when MJ produced no response, or an incorrect response, one of the experimenters then gave her up to two verbal cues. Firstly, if a correct response had not been produced, the experimenter gave MJ a semantic cue: either the name of the superordinate category of the pictured object, or a functional feature of the object. Semantic cues were entirely ineffective in aiding her performance. Secondly, the experimenter gave MJ a phonemic cue, consisting of the first two phonemes of the target picture name. Remarkably, the phonemic cues helped MJ to produce a correct answer in 31 cases where previously she had produced an incorrect picture name, or had been wholly unable to produce any response. The targets that resisted cueing in nine cases elicited no response, and in four cases elicited related errors. The related errors consisted of, in one case, a semantically and phonologically related word, ZANCOS (stilt) → <<zancas>> (shanks), in one case, a phonologically related word, ARPA (harp) → <<arpón>> (harpoon), and in the remaining two cases, phonologically related nonwords, FONENDOSCOPIO (stethoscope) → <<fonunculo>> and CHUPETE (pacifier or dummy) → <<chupeta>>. We have seen that MJ's semantic knowledge appears to be intact. Together, the observations reported in previous sections, and these cueing data, support the view that MJ's speech production problems may arise at the phonological level, or at the connection between semantics and phonology (please see Table 1).

Table 1
Table showing performance in tests of word recognition, comprehension and semantic knowledge

| Source | Test | | Number of items | Responses correct | Percentage correct (%) |
|---|---|---|---|---|---|
| EPLA | visual lexical decision | | 40 | 36 | 90 |
| EPLA | auditory word-to-picture matching | | 40 | 38 | 95 |
| study-specific | close semantic relatives discrimination | | 40 | 40 | 100 |
| study-specific | Spanish ‘Pyramids and Palm Trees’ | | 25 | 24 | 96 |
| EPLA | synonym judgment test | total | 60 | 53 | 88.33 |
| | | high imageability* | 30 | 29 | 96.67 |
| | | low imageability* | 30 | 24 | 80.00 |
| study-specific | fluency | fruits | 2 | | |
| | | animals | 4 | | |
| | | p | 1 | | |
| | | f | 0 | | |
| RBNT | picture naming | total | 60 | 16 | 26.67 |
| | | phonemic prime* | 44 | 31 | 70.45 |

Note: EPLA is an abbreviation of ‘Evaluacíon del Procesamiento Lingüístico en La Afasia’, by Valle and Cuetos [47], RBNT is an abbreviation of the Revised Boston Naming Test [26].
*For the synonyms judgment test, we show both the total number of responses correct, and the number correct to 30 high imageability or 30 low imageability word pairs. For the picture naming test, we show the total number of responses correct out of 60, then the number of responses successfully cued by a phonemic cue out of 44, the number of previously incorrect responses.

### 2.4. *Word and non-word repetition*

MJ has substantial word finding difficulties in spoken production. Was it the case that MJ was unable to produce picture names, or word associates because she could not produce the phonological form of the target words? We examined MJ’s output phonology in tasks that did not entail semantic processing, by testing her ability to repeat spoken words and nonwords.

The first task was extracted from the EPLA. MJ was asked to repeat aloud 24 words and 24 nonwords. The items varied from three to six letters in length, with six words and six nonwords presented under each length condition. The nonword targets had been created by substituting one phoneme in each of a selection of words to create 2-syllable pronounceable phoneme strings. In the word repetition task, we found that MJ was able to correctly repeat 23 out of 24 words spoken to her (95.83%). Her only error was in the repetition of a three-letter word, <<tía>>. In the nonword repetition task, MJ was able to repeat 21 out of 24 target nonwords spoken to her (87.5%). One of her errors was the production of a phonologically related word, <<iro>> → <<ido>> (absent-minded), the other two incorrect responses were phonologically related nonwords, <<blodia>> → <<rodria>>, <carbe>> → <<cansel>>, and <<dombra>> → <<rombra>>. Normative data reported for the EPLA indicates that MJ’s repetition of words and nonwords was normal. Healthy adults can be expected to produce errors sometimes even to three-letter words (on average, scoring 5.91 correct out of six on three-letter words, s.d. of 0.29). They can also be expected to repeat correctly, on average, 5.73 out of six five-letter nonwords (s.d. of 0.86) and 5.36 out of six six-letter nonwords (s.d. of 1.19).

MJ’s performance in the nonword repetition task suggested a slight impairment of her capacity to create new phonological forms for output. To investigate this possibility further, we created a new test in which we asked her to repeat 12 nonwords, varying in length from four to eight phonemes. She was correct on 11 items (91.67%). This observation, together with her performance in the first test suggests that MJ was unimpaired in her ability to produce speech.

### 2.5. *Nonword reading*

We probed MJ’s ability to read nonwords aloud in two tests. In the first, we presented a set of 40 nonwords. 24 had been extracted from a nonword reading test in the EPLA, and varied in length from three to six letters. 16 items were added to increase the range of length variation to eight letters. All items were pronounceable letter strings created by substituting one or two letters in real words. MJ was able to produce only two correct responses in this task (5% of 40 nonwords). For 23 items (57.5%), she produced no response at all. For nine items (22.5%), she produced a nonword response e.g. “jia” → <<jira>>. For six items (15%), MJ produced a lexicalization response, e.g. “carbe” → <<carbon>>, phonologically related to the target, where phonological relatedness is attributed

Table 2
Table showing performance in tests of phonological output

| Source | Test | Number of items | Responses correct | Percentage correct (%) |
|---|---|---|---|---|
| EPLA | auditory word repetition | 24 | 23 | 95.83 |
| EPLA | auditory nonword repetition 1 | 24 | 21 | 87.50 |
| study-specific | auditory nonword repetition 2 | 12 | 11 | 91.67 |
| study-specific | pure nonwords reading aloud | 40 | 2 | 5.00 |
| EPLA | mixed words and nonwords: nonword reading | 20 | 0 | 0.00 |
| EPLA | mixed words and nonwords: word reading | 20 | 9 | 45.00 |

if the response shares 50% or more of its phonemes with the target. It is apparent that MJ's performance in this first test is severely impaired. (No normative data are directly applicable to the whole test, but for the 24 items taken from the EPLA one could expect healthy adults to have achieved, at minimum, 19 items correct).

In the second test, we asked MJ to read a mixed list of 20 words and 20 nonwords, extracted from the EPLA. The nonwords were created in the same manner as those employed in the first test. MJ was able to read nine of 20 words correctly (45% of the words); we discuss the errors produced to words further in the section on word reading. She produced no correct responses to the nonwords but she produced five lexicalization errors, "asbol" → <<árbol>> (tree), "hospitel" → <<hospital>> (hospital), "fueco" → <<fuego>> (fire), "ajercipo" → <<ajedrez>> (chess) and "obredo" → <<obrero>> (working), while for all other items she was unable to produce any response.

The evidence yielded in these tests suggests that MJ cannot assemble the phonology required to read aloud letter strings that are not part of her lexicon, though she can repeat auditory nonwords (please see Table 2). MJ's inability to assemble phonological forms in response to nonword letter strings cannot easily be argued as being due to a problem of visual perception because MJ is perfectly able to distinguish words from nonwords in a visual lexical decision task. We suggest that MJ's nonword reading impairment could be caused either by an impairment of knowledge about grapheme-phoneme correspondences, or by an impairment of the ability to assemble the outputs of such correspondences.

### 2.6. *Sound-letter matching, letter naming, and measures of short-term memory span*

We probed directly MJ's capacity to make mappings between phonemes and graphemes. In a test of letter-sound matching, extracted from the EPLA, the experimenter said aloud 24 of the letter sounds of the Spanish alphabet, in series in random order, and then asked MJ to point out which of four printed letters corresponded to the sound. The set of candidates consisted of the target letter corresponding to the sound, an unrelated letter, and visually or phonologically related letters. MJ was correct in all 24 trials.

In a test of letter naming, also extracted from the ELPA, MJ was given 26 letters of the Spanish alphabet, in random order, and was asked to either say their name or give the sounds with which they could be pronounced e.g. for "r" the name <<erre>> or the sound /re/. We found that she was able to produce 20 correct responses in 26 trials (76.92%), which is impaired relative to the lower limit of normal performance, estimated at 25.54 correct for healthy adults. The correct responses were a mix of letter names and letter sounds. MJ could not produce correct responses to the letters "l, x, n, h, y, [and] f". Inspection of MJ's errors suggests that she was impaired by a word finding difficulty similar to the problem she presents in the picture naming task. One error arises from a seeming visual confusion, when MJ names "l" at first as <<uno>> (one). Another possible visual error may account for her production of the letter name <<eñe>> (for "ñ") to the item "n", but this is as likely a perseverative error since the letter "ñ" (to which MJ responded correctly) appeared before the letter "n" in the test. In two cases, in her responses to "f" and "x", MJ was incorrect because she attempted to find the correct name by rehearsing the letters of the alphabet yet she settled on the wrong letters, her errors being, respectively, <<ka>> and <<erre>>. We suggest that the latter errors reflect a strategy to compensate for a word finding difficulty. This hypothesized lexicalization problem may have been particularly acute in two further cases, where MJ produced a fragmentary response, <<che>> (/ʃe/), to one letter "h" which is silent but has a name, <<ache>>, and produced no response to another letter, "y", which has a sound identical to that for "i" but a difficult name, <<igriega>>.

We examined MJ's span for both numbers (the numbers 0 to 9) and concrete bisyllabic high frequency words. In both tests, MJ was asked to repeat strings of items immediately after one of the experimenters said

Table 3
Table showing performance in tests of grapheme-phoneme correspondences and of short-term memory span

| Source | Test | Number of items | Responses correct | Percentage correct (%) |
|---|---|---|---|---|
| EPLA | letter-sound matching | 24 | 24 | 100.00 |
| EPLA | letter naming | 26 | 20 | 76.92 |
| study-specific | verbal span | 2 | | |
| study-specific | digit span | 2 | | |

them aloud. A test was discontinued at the presumed limit of MJ's span when she as unable to repeat, on two consecutive occasions a string of items of that length. MJ was found to have a digit span of three numbers, and a verbal span of three words.

We suppose that MJ must be able to achieve mappings from graphemes to phonemes to match a target letter sound to the appropriate letter. Evidence gathered in the letter naming task suggests, however, some impairment of her ability to produce phonemes to target graphemes (please see Table 3). This problem could reflect an impairment of MJ's knowledge of grapheme-phoneme correspondences, or of her ability to complete such correspondences, revealed in a task that is more difficult because it requires production rather than matching. We consider that her errors in the letter naming task could equally be seen, however, as further evidence of a word finding difficulty. Thus, it may be more revealing of the causes of MJ's impairments that she presents a severely restricted short-term memory span. Such a restriction could be expected to critically weaken her capacity to assemble the outputs of grapheme-phoneme or phoneme-grapheme correspondences.

### *2.7. Reading words aloud*

In the section reporting MJ's ability to read nonwords, we saw that in a test in which words and nonwords were presented to her in a mixed list she could read only nine correct out of 20 words. In the present section, we report a series of tests designed to elucidate in detail the characteristics of MJ's reading abilities. We report the outcome, firstly, of each individual test before discussing the pattern of correct and incorrect responses yielded across all tests. (An analysis of MJ's performance, giving the total number of correct responses in each test, and the total correct within each condition, is shown in Table 4.)

In a test extracted from the ELPA, we probed the effects of imageability and frequency on MJ's oral reading. We asked her to read aloud 80 words selected to vary in relation to imageability and frequency in a factorial design. MJ read aloud correctly 28 (35%) of these words. Of those words that she produced correctly, more were highly imageable than were of low imageability, and more were of high frequency than were of low frequency. Chi-square tests indicate that these differences were significant (test of effect of imageability, chi-sq. $= 13.81$, 1d.f., $p < 0.01$; test of effect of frequency, chi-sq. $= 4.8$, d.f. $= 1$, $p < 0.05$).

In a second test, also extracted from the ELPA, we examined the effect of the grammatical class of words on MJ's ability to read them aloud. We presented 80 items consisting of 20 items from each of the classes: nouns, verbs, adjectives and function words. Words were matched across grammatical class on frequency and imageability. MJ was able to read 17 items correctly (21.25% of the total). We found that she was able to read more nouns correctly than verbs, adjectives or function words. A test of the differences between reading accuracies for each class did not indicate that the differences are significant, but we note that the overall level of performance is relatively low so that performance under each condition may have been at floor, diminishing the likelihood of observing potentially substantial length effects.

In a test of the effect of length, developed for the study, we asked MJ to read aloud 30 words varying in length in letters. Short words were 3–5 letters in length while long words were 7–13 letters in length. Words were matched for frequency across length conditions. We found a small effect of length, MJ read correctly more short words (five out of 15) than long words (one correct) but clearly her performance is very low across length conditions. She only produced six correct answers (20% of the total) so that her performance might be considered to be at floor.

We examined the effect of regularity on MJ's reading by presenting a set of 18 words borrowed from English, in common use in Spanish. These words are pronounced in a manner inconsistent with Spanish grapheme-phoneme correspondences, so that e.g. "Hollywood" is correctly read as /holiwud/ but incorrectly

Table 4
Table showing performance in tests of oral reading

| Source | Test | Number of items | Responses correct | Percentage correct (%) |
|---|---|---|---|---|
| EPLA | imageability x frequency total | 80 | 28 | 35.00 |
| | high imageability* | 40 | 22 | |
| | low imageability | 40 | 6 | |
| | high frequency | 40 | 18 | |
| | low frequency | 40 | 10 | |
| EPLA | grammatical class total | 80 | 17 | 21.25 |
| | nouns | 20 | 7 | |
| | verbs | 20 | 5 | |
| | adjectives | 20 | 2 | |
| | function words | 20 | 3 | |
| study-specific | length total | 30 | 6 | 20.00 |
| | short | 15 | 1 | |
| | long | 15 | 5 | |
| study-specific | regularity | 18 | 5 | 27.78 |
| study-specific | pure nonwords reading aloud | 40 | 2 | 5.00 |
| EPLA | mixed words and nonwords: nonwords | 20 | 0 | 0.00 |
| EPLA | mixed words and nonwords: words | 20 | 9 | 45.00 |

Note: the nonword data is included here for easy comparison.
*The entries for the imageability x frequency test shows the number of responses correct in total, then for each condition firstly, showing performance on those words that are high or are low imageability in the set of 80, then showing performance on those words that are high or low frequency.
Likewise, the entries for the grammatical class test show performance for all items, then for items by grammatical class, and the entries for the length test show performance in total, then by condition.

as /ojibood/: the first reading is consistent with the pronunciation in English; the second is the regular Spanish pronunciation for the letter string. MJ read five of these irregular words correctly (27.78% of the total). For six items, MJ produced no response. Her errors suggest, however, that the low level of her performance need not be ascribed to a low level of general knowledge since for six items she produced a semantically related word e.g. “hollywood” → <<fiesta>> (party), or a circumlocution e.g. “renault” → <<run-run, Schumacher, Alonso>>. She produced a regularization error in one case only, pronouncing “pub” as /puf/, which can be presumed to indicate that she retained some minimal use of grapheme-phoneme correspondences.

We have seen that MJ is able to read a proportion of the words given to her in tests of reading aloud. Across the tests, the level of her performance is relatively low, though not as low as, for example, JMK’s performance, discussed in the Introduction. We suggest that to some extent this low level of performance is attributable to the consequences of matching, for example, words across grammatical classes. Such matching is required for comparisons between classes but will tend to drive the frequency or imageability of words down to the lowest level common between classes. There are clear-cut effects of frequency and imageability that suggest that, as a result, MJ’s performance in oral reading of words may be better, overall, than the tests reported indicate – she could be expected to present better performance on words sampled from the middle and higher end of the frequency range.

An examination of the types of incorrect responses produced in all tests of reading (see Table 5) suggests the lexicality of MJ’s reading procedure. She read 65 (28.51%) words correctly out of a total of 228. For 72 (31.58% of the total number) items she made no response. Critically, she produced a number of verbal paralexias. She produced 23 semantic paralexias, e.g. for the target “ventana” (window) she produced the semantically related <<puerta>> (door). She also produced circumlocutions, explaining the meaning of words she could not name, for eight items e.g. “disjockey” → <<fiesta, no parecido>> (party, no, similar). 26 of her responses were related only by visual similarity to the target words, e.g. “libre” (free) → <<libro>> (book). Three of her responses can be interpreted as having been produced through, firstly, a visual, and then secondly, a semantic error. That is, we suppose that e.g. the stimulus “momento” (moment) elicits the response <<iglesia>> (church) via, firstly, a visual error, mistaking “momento” for the semantically unrelated “monumento” (monument), secondly, confusing the semantic relatives monumento and iglesia. MJ produced 13 morphological errors e.g. substituting the feminine with

Table 5
The number and percentage of correct and error responses (of all types) observed in tests of reading words aloud

| Response types | Number | Percentage of total (%) |
|---|---|---|
| correct | 65 | 28.51 |
| omission | 72 | 31.58 |
| semantic | 23 | 10.09 |
| visual | 26 | 11.4 |
| visual-then-semantic | 3 | 1.32 |
| morphological | 13 | 5.7 |
| circumlocutions | 8 | 3.5 |
| function word substitutions | 1 | 0.44 |
| nonwords | 2 | 0.88 |
| unrelated | 15 | 6.58 |
| total | 228 | 100 |

Table 6
Table showing performance in tests of spelling to dictation

| Source | Test | Number of items | Responses correct | Percentage correct (%) |
|---|---|---|---|---|
| EPLA | regular words | 20 | 6 | 30.00 |
| | irregular words | 20 | 5 | 25.00 |
| EPLA | nonwords | 24 | 3 | 12.50 |

the masculine suffix in producing <<nuestro>> in response to “nuestra” (ours). Two of her errors were nonwords e.g. “pub” → <<puf>>. 15 errors were words unrelated to the target letter string e.g. “atención” (attention) → <<abogado>> (lawyer). We would argue that the pattern of errors indicate that MJ produced responses by first accessing semantics, through mappings from graphemes to orthographic representations, and then deriving phonological words from semantic specifications.

### *2.8. Spelling words and nonwords to dictation*

MJ adopted her left hand for writing, following her injury, though previously she had favoured her right hand. Despite this, she was well able to form letters so that it seemed likely that any errors recorded in tests of writing would reflect cognitive linguistic rather than motor control factors. We first tested MJ’s ability to spell words to dictation, using a test extracted from the EPLA. We presented a mixed list of 40 items, 20 regularly spelled words and 20 words containing phonemes with inconsistent spellings. MJ produced 11 correct spellings (27.5% of the total). We found no effect of regularity on MJ’s spelling since she correctly produced spellings for six regular and five irregular words. Healthy adults could be expected to spell, on average, 19.36 regular words (s.d. of 1.37) and 16.23 irregular words (s.d. of 4.09) correctly. For five items, she produced no response at all. Her errors included four words orthographically and (necessarily) phonologically related to the target e.g. “verso” (line) → “verbo” (verb); where a formal relation is attributed if the response shares at least 50% of letters with the target, in any order. The remainder of her errors consisted of nonwords orthographically and phonologically related to the target e.g. “almohada” (pillow) → “alroada”.

We tested MJ’s ability, next, to spell nonwords to dictation. Using 24 items taken from the EPLA, consisting of word-like sounds formed by substituting one phoneme in real words, we found that MJ could produce only three correct spellings (12.5%). She did not omit to produce a response, but the rest of her responses consisted of errors that, in six cases, were words orthographically and phonologically related to the targets e.g. “siplo” → “sapo” (toad), in 14 cases consisted of nonwords similarly related to the targets e.g. “uto” → “ote”, and, in one case, a nonword unrelated to the target “valo” → “tana”.

## 3. Discussion

MJ suffers severe impairments of spoken and written word production. She can read a minority of the words given to her in tests of reading aloud but she is often unable to produce a response, or the responses that she can produce are often incorrect. Her reading errors are characterized predominantly by semantic, visual, or derivational similarity to the target words. She is clearly much worse at reading words that are less imageable, or less frequent. And she presents a numeric trend in being worse at reading verbs than nouns or adjectives. Her spelling to dictation is, also, very impaired. She is unable to read aloud nonwords, and is very poor at spelling them. She has a severely restricted verbal short-term memory span. In all these respects, MJ presents a pattern of preserved and impaired abilities characterized, in studies of reading in English, as deep dyslexic.

Yet MJ demonstrates substantial preservation of her semantic knowledge, as well as a preservation of her abilities in visual word recognition and spoken word comprehension. That her semantic memory is preserved is underlined by the fact that she can often mime the meanings of words she cannot read. In addition, her word-picture matching and her performance in tasks probing detailed semantic relations was at a high level. Furthermore, MJ does not present an impairment of her capacity for phonological output for she is able to repeat spoken words and most nonwords. In sum, the evidence supports the conclusion that her difficulties stem primarily from a problem of lexical selection for output.

MJ's inability to read nonwords suggests that she is unable to use grapheme-phoneme correspondences to read aloud. The reasons for this deficit may be twofold. Her ability to perform phoneme-grapheme matching suggests that she retains the capacity to distinguish appropriate or inappropriate grapheme-phoneme correspondences. But her relatively poor performance in the letter naming task indicates that she is impaired in her ability to perform such correspondences for output. The second reason for her impaired nonword reading may relate to the fact that MJ demonstrates a severely restricted short-term memory span. We would argue that this restriction would weaken MJ's capacity to blend the outputs of those correspondences she can complete. Together, the problems we have highlighted would result in her nonword reading impairment.

An inability to use sub-lexical correspondences would force MJ to rely solely on direct lexical mappings, via semantics, to read aloud. We assume that in the normal reading of healthy adults, a reader can use both routes to read. We suppose that MJ employs lexical mappings to read words because the degree of success she shows in reading is clearly affected by lexical properties such as imageability. In addition, the errors that she makes embody lexical qualities. Above all, the semantic relatedness of some errors, the circumlocutions produced to some target words, and her capacity in many cases to mime the meaning of words she could not name, reveal a semantically mediated reading strategy.

The question then arises, why is her reading impaired, if she may rely on lexical mappings? We suggest that MJ suffers a lexical selection deficit. In our review, we discuss the idea [43] that semantic paralexias may occur in the reading of deep dyslexic patients because, variously, access to semantics, semantic processing, or phonological output may be damaged. We have shown that MJ's visual word recognition ability, her semantic knowledge, together with her comprehension abilities, as well as her phonological output capacity, appear to be well preserved. In addition, she demonstrates a fine awareness of the semantic errors she produces when she reads. Finally, the finding that MJ's picture naming was markedly aided by phonemic cueing indicates not that MJ has 'lost' words but that she has difficulty appropriately bringing the phonological forms for words she knows to the point of output.

It has been suggested that the loss of the outputs of grapheme-phoneme correspondences (GPCs) can lead to the impairment of procedures whereby the outputs of direct lexical mappings are edited using the outputs of GPCs [34]. One reason that direct lexical mappings may need editing is that the semantic specifications of some words, especially words low in imageability, may not be sufficiently precise to deliver appropriate words for output. We accept that, in reading, the advantage associated with words of high imageability suggests that more imageable words are easier to lexicalize. There is an imageability effect in MJ's reading but she is well able to perform a semantic processing task like synonym judgment even for low imageability words. Further, as noted, MJ demonstrated an acute awareness of her semantic errors in reading, suggesting the capacity for semantics to distinguish appropriate lexical outputs. It is unclear to what extent MJ is impaired in her capacity to effect grapheme-phoneme correspondences. She cannot use their outputs to read nonwords aloud. She shows some impairment of her capacity to perform sub-lexical mappings, but she retains sufficient knowledge to distinguish letter-sound matches. On balance, we would argue that MJ could receive some weakened help from her knowledge of grapheme-phoneme correspondences but that either it is not enough to prevent the production of verbal paralexias or it is sufficient for such a purpose, but is not, in fact, normatively used to edit candidate, semantically specified, reading outputs.

We note that in the mixed word and nonword reading test, MJ produced only lexicalization errors or produced no responses in reaction to nonword items, whereas in the pure nonword reading test she did produce some non-lexical responses. Baluch and Besner [5] argue that the admixture of nonwords to a test of reading words aloud promotes the use of sub-lexical reading strategies by readers of a transparent orthography, Persian. We cannot say if the words in the mixed word/nonwords reading test were read in a more sub-lexical fashion, given the small number of relevant data points. There is a suggestion in the lack of nonword incorrect responses in MJ's reading of the (mixed list) nonwords that where she expects some or all the stimuli to be legitimate words then she uses residual knowledge of grapheme-phoneme correspondences to edit her responses. She may use the knowledge to block the selection of words that do not fit both the visual and (perhaps impaired) phonological specifications. In comparison, in the pure list nonword reading test, where she does not expect to see legitimate words, she allows herself to produce nonword responses, albeit in only a small number of instances.

We would argue, therefore, that the use of some grapheme-phoneme knowledge may help to account for the occurrence of omissions in MJ's responses but that such usage could be sufficient, if granted, to nullify the production of semantic paralexias. Yet such errors are observed. Thus we believe that a more convincing explanation for the occurrence of semantic paralexias is that they stem from a problem of lexical selection. Such a problem could well be linked to her short-term memory deficit. In recent researches conducted with healthy adults, experiments designed to probe the lexical selection mechanism in speech production have yielded findings that can be argued to show that lexical selection requires the activation of lexical candidates for output and their abstraction for selection to working memory. The critical observation in these studies is that if the set of potential candidates for selection exceeds the hypothesized capacity of short-term memory then the field of candidates appears to be limited only by semantic relatedness, whereas task relevance might otherwise also serve to constrain it. The experiments have been conducted using the picture naming task [8,9,38] but the principle applies whether we consider semantically-driven speech production in picture naming, or, as argued here, semantically driven production in deep dyslexic reading. MJ's severely restricted short-term memory span can be seen, with respect to these findings, to render her vulnerable to greatly increased competition for selection between lexical candidates for speech production. Such a vulnerability could be expressed in semantic paralexias even if some phonological knowledge could be used to edit outputs.

If we now consider the evidence gathered in relation to MJ, together with that reported concerning the other cases reviewed in this paper, we believe that in sum it urges the conclusion that direct lexical mappings are used in reading in Spanish. It has been argued that for readers of transparent languages the process of reading depends entirely on sub-lexical mappings between graphemes and phonemes ([1–3], though see [37,39] for counter-arguments). Such mappings are employed when words have to be read aloud. The same mappings are employed when reading is for understanding: the reader contacts the lexicon, and so accesses semantics, by mapping graphemes to phonemes to obtain the phonologies of the words being read. In the strongest versions of this account ([1], see also [22]) the route to phonology or to meaning is always and only through grapheme-phoneme correspondences. This contrasts with the system envisaged for readers of languages with quasi-regular orthographies like English, where the irregularities of correspondences warrant the adoption of direct lexical mappings. Why would readers of a highly transparent alphabet, like that used in Spanish, adopt such mappings? We think it plausible that, even if an individual must read words printed in a transparent alphabet, high levels of literacy promote direct lexical mappings as a quick or efficient route to the lexicon.

How skilled do you have to be to have developed such mappings? How much print exposure is required to promote such development? Print exposure is difficult to quantify and thus the developmental links between exposure and the formation of direct lexical mappings is uncertain. It can be noted, however, that Landauer and Dumais [30] discuss data which suggests that vocabulary expands with very great rapidity in childhood, and report simulations of their own which, together with the developmental data, indicates that semantic representations for words can be acquired purely by exposure to print, and the statistical distribution of lexical co-occurrences.

Consideration of these questions is linked to the related question, could the reading of the patients discussed in the present article be ascribed to the therapy they received? On balance, we contend that the high premorbid literacy of the patients strikes would afford a stronger basis for direct lexical mappings, evidenced in all case studies, than the therapy they received, which varies in amount and kind across individuals.

We turn, at last, to the issue of why so few cases of deep or of phonological dyslexia in Spanish have been reported, compared with the number of cases reported in English or in French. We suggest that the paucity of such cases can be attributed largely to sampling factors. Cases of acquired deep or phonological dyslexia must, of course, be expected to occur with great rarity, relative to the number of readers in any language population. Moreover, awareness of the deep dyslexias among authorities capable of referring patients for study can be supposed to be greater in the United Kingdom, France, or the United States, where much of the research investigating the dyslexias has been conducted, than it is at present the case in Spain or, indeed, in the global Spanish-speaking community. In addition, we note that the patients presenting symptoms of deep or phonological dyslexia that have been reported in Spain have all been relatively young, relatively well educated individuals. Yet the occurrence of lesions that lead to central impairments of reading would tend to be seen more often in older patients. It is important to acknowledge that the civil war and postwar period in Spain disrupted the education of a generation of individuals who are now at or approaching the age at which there might be greater likelihood of the occurrence of the lesions that inflict central reading impairments. Thus it may be that the rarity of such lesions, combined with lower levels of literacy in the individuals in whom such lesions might be more likely to occur, would tend to drive down the number of cases of deep or phonological dyslexia observed.

## Acknowledgements

We owe profound thanks to MJ for her participation in our study. The research reported, and the writing of the report, was supported by the Marie Curie Foundation (grant number UE-05-MRTM-CT-2004-512141).
